# Explainer: Extaille

**DOI:** 10.1017/ext.2026.10022

**Published:** 2026-09-28

**Authors:** J. Michael Reed

**Affiliations:** Department of Biology, Tufts University, USA

**Keywords:** abundance, conservation status, decline, ecosystem function, functional extinction

## Abstract

When describing the status of a species, extant and extinct are well defined: ‘existing’, and ‘no longer in existence’, respectively. What is the appropriate term, however, for a species that is extant, but whose abundance has dropped to an exceptionally low value? In practice, we tend to describe this species as ‘nearly extinct’, or similar. Terms such as ‘rare’ and ‘endangered’ have multiple meanings, but none address ecological functions. Also, in conservation biology, when we ask ‘what drove a species extinct?’, we do not mean ‘what killed the last individual?’ – we mean ‘what drove a species’ population size so low that extinction was likely?’. There is no term for this, so we rely on imprecise wording and assume that the reader knows what we mean. Banks proposed the term ‘extaille’ to fill this void (pronounced ex-tayl). Extaille is the status of a species whose abundance has been reduced so much that it no longer functions as a significant part of its normal ecosystem; “extation” refers to the condition of becoming extaille. These terms have not caught on in the scientific literature, and I recommend against them. Use of this jargon could hurt communication outside the discipline, and a term like “functionally extinct” (or similar) better meets our needs.

## Graphical abstract

**Figure:**
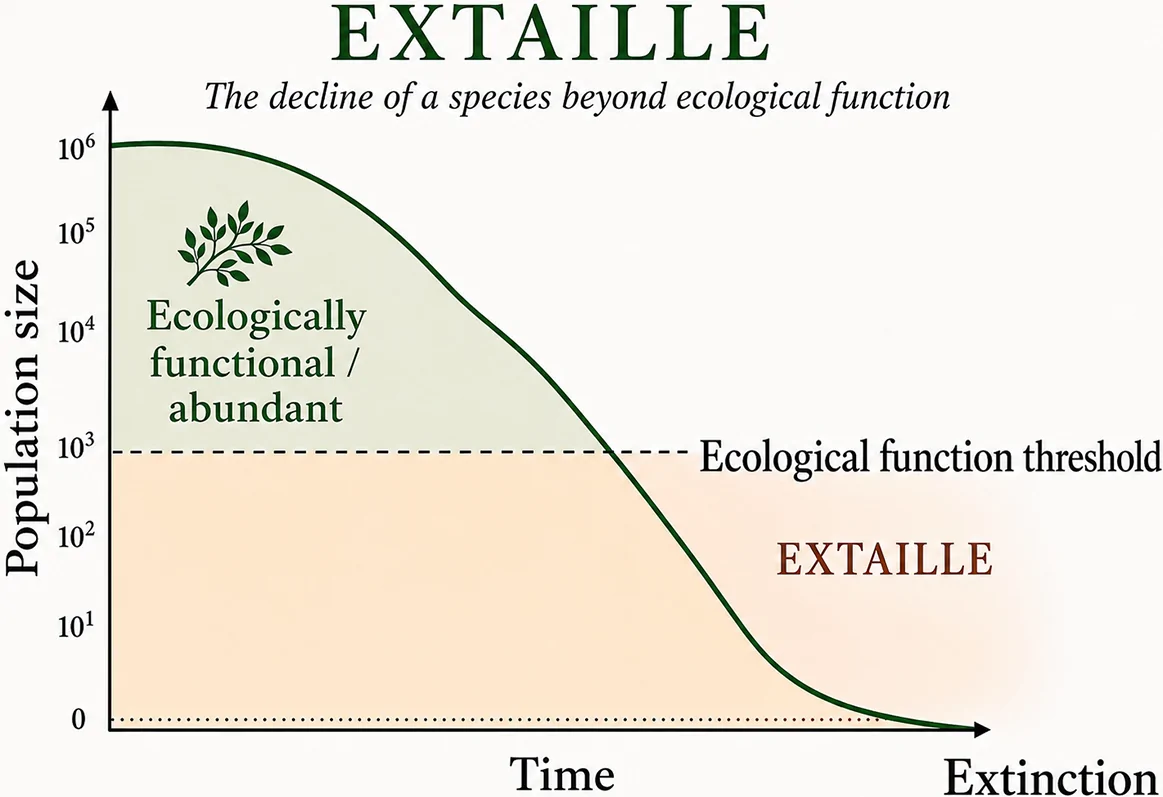

## Impact statement

Because there was no term for a species or population that had declined so much that it was no longer ecologically relevant, Banks proposed the term extaille. Banks also thought this term would clarify the question of what drove a species extinct – because what researchers typically mean is ‘What drove a species to its very low population size?’. This term, however, never caught on. Instead, we now use less jargony terms like “functionally extinct” to describe this species’ status.

## Background and assessment

As part of the lexicon of conservation biology terms, we have a suite of ‘ex-’ words to describe the status of populations: extant, extirpated and extinct. These refer to species that exist, local loss of populations or global loss of a species, respectively. Extant, however, encompasses everything from billions of individuals to a single individual, so it tells nothing about the status of a species.

Population size can decline through deterministic forces, and it can eventually become sufficiently uncommon that it loses ecological functionality and might eventually drop into an extinction vortex (Soulé et al., 2005; Mills et al., 2012). Using population or species functionality as a metric in conservation is not new. Not long after the concept of minimum viable population size was introduced (Shaffer, 1981), Conner (1988) proposed ecological functionality as a definition of viability. More recently, Reed and McCoy (2025) proposed a definition of ‘small’ population size based on functionality.

How might one describe the process of population decline to the point of no longer being ecologically significant? Terms such as ‘rare’ and ‘endangered’ have multiple meanings focusing mostly on population size and extinction risk, but none address ecological functions (Rabinowitz, 1981; Reed et al., 2020; IUCN, 2024). This is where Banks (1976) stepped in, proposing a new ‘ext-’ term: extaille. In a letter to *Science*, he laid out the case for the term.

**Figure 1.**
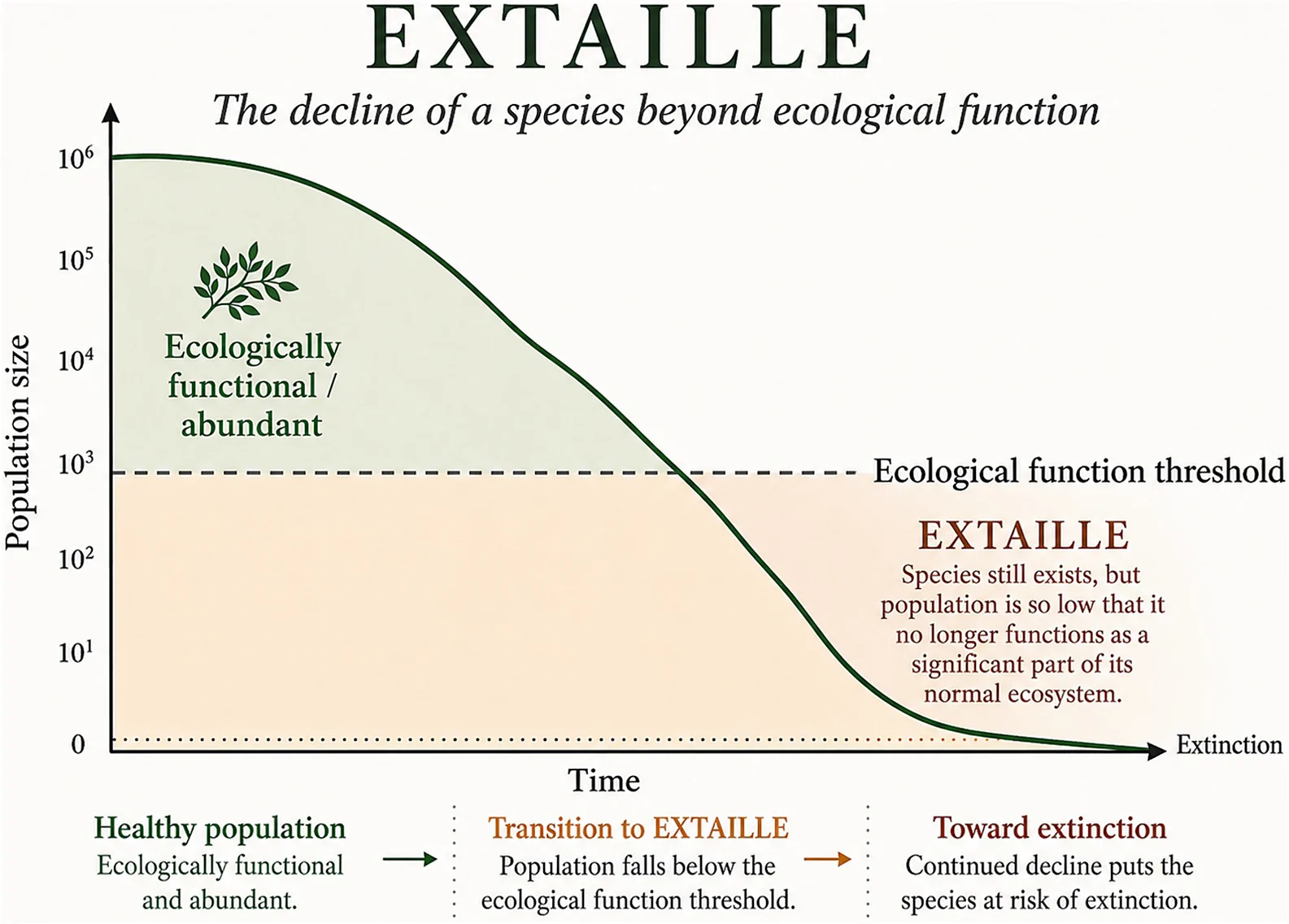
A graphical depiction of a species going extaille. Numbers on the y-axis are just for example, and the threshold would differ depending on your focus (e.g., ecosystem function and ecological service). I used ChatGPT Plus interactively to create this figure and the graphical abstract. Figure 1. long description.

Banks stated that terms like ‘almost extinct’ or ‘nearly extinct’ were vague, conveyed little about the status of a species, and because extinct or not is a binary state, an ambiguous in-between was not useful. He proposed extaille from ‘taille’, meaning cut or trimmed, suggesting a ‘remnant from a formally abundant population’ (pp. 1217 + 1292) that was too uncommon to serve its ecological functions (Figure 1). The ‘ex’ prefix was added to be in line with extant and extinct. Extation is the condition of being extaille. When one asks ‘What drove a species extinct?’, what we really mean is ‘A species is extaille – what drove it to extation?’

These terms (extaille and extation) were never established in the academic literature. They were used twice in the scientific literature the year following Banks’ letter. Banks (1977) used the term in his review of the decline of the Eskimo Curlew (*Numenius borealis*). The traditional story is that this species was driven extaille by overharvest from market hunters. Banks speculated that the decline started before market hunting was excessive, and that climate change altering storm patterns that affected migration might also have contributed to the decline. That same year, Le Boeuf (1977) proposed that Elephant Seals (*Mirounga augustirostris*) had recovered from extation. I did not find the term used again for over 30 years (Elphick et al., 2010). More recently, there has been a small uptick in its use, but only in passing or listed as a keyword (Le Boeuf, 2021; Martin et al., 2023; McCrea et al., 2024).

Should the term extaille be in more common use in the conservation literature? One might argue for its adoption because of its precise definition, but I see it inhibiting communication in cross-disciplinary work, and it would make communication with the public more difficult. Conservation science focuses on the process of population collapse (Caughley, 1994), so when one asks ‘What drove a species extinct?’, they never really mean ‘what killed the last individual?’, so extaille is not needed to resolve this potential issue. Alternatively, I suggest that using a term such as ‘functionally extinct’ (or similar), combined with a specific description of the function(s) of interest that have been lost (Säterberg et al., 2013; Jarić, 2015), better serves our needs. This resolves Banks’ concerns, and it reduces confusing created by jargon.

## Data Availability

There are no data in this paper.

## Long descriptions

### Figure 1. Long description

The graph features a vertical y-axis labeled Population size with a logarithmic scale ranging from 0 to 10 super 6. The horizontal x-axis is labeled Time, ending at a point marked Extinction. A dark green sigmoidal curve begins at the top left at 10 super 6, remains stable briefly, then descends steeply toward the bottom right.

A horizontal dashed line at the 10 super 3 mark is labeled Ecological function threshold. This line divides the graph into two colored zones.

1. The upper zone, shaded light green, is labeled Ecologically functional / abundant and includes a small icon of a leafy branch.

2. The lower zone, shaded light orange, is labeled EXTAILLE in large brown capital letters.

The curve crosses the threshold line at approximately the midpoint of the time axis, moving from the green functional zone into the orange EXTAILLE zone before finally reaching zero at the extinction point. The subtitle at the top reads: The decline of a species beyond ecological function.

Navigate back to Figure 1..
