# Supplementary material for: Explainer: Extaille
Source: Camb Prism Extinct. 2026 Sep 28;4:e13. doi: 10.1017/ext.2026.10022 (PMC13748985; doi:10.1017/ext.2026.10022)
Supplement: Peer review history

# Reviewer report: Review: Explainer: Extaille — R0/PR2

**DOI:** 10.1017/ext.2026.10022.pr2

I agree with the author. There is a need for a term that describes a species that is virtually extinct. The Banks term, extation, did that but it didn’t catch on. We need a term that is understandable across disciplines. A better term might be, functionally extinct.

# Reviewer report: Review: Explainer: Extaille — R0/PR3

**DOI:** 10.1017/ext.2026.10022.pr3

**Authors:** Hui Zhang

I generally support the main logic and final recommendations of this paper, but suggest that qualifying conditions be added to certain statements:

1. The binary classification of species as ‘extant’ or ‘extinct’ contains gaps in its formulation; the terms ‘rare’ and ‘endangered’ focus on population size and the risk of extinction, but lack the dimension of ecological function; conservation science focuses on the process of population collapse rather than the death of the last individual, and I agree with this assertion.

2. The concept of ‘extaille’, coined by Banks, precisely addresses a scientific gap; however, as it lacks academic consensus and hinders interdisciplinary communication, its widespread adoption is not recommended.

3. Within the existing, well-established terminological framework, ‘functional extinction’ is the most appropriate term to describe a situation where ‘only a small number of individuals remain, population abundance is extremely low, and the species has lost its original ecological function’.

4. When using the term ‘functional extinction’, one should proactively and explicitly define its operational definition within papers or texts to compensate for the lack of a unified quantitative standard for this term and to mitigate its inherent precision limitations.

# Editor report: Recommendation: Explainer: Extaille — R0/PR4

**DOI:** 10.1017/ext.2026.10022.pr4

**Affiliations:** Durrell Institute of Conservation and Ecology, University of Kent, United Kingdom of Great Britain and Northern Ireland

Having read the manuscript and received two very positive reviews only some very minor corrections/clarifications have been suggested to be considered. I believe this should be very quick to resolve.

# Editor report: Decision: Explainer: Extaille — R0/PR5

**DOI:** 10.1017/ext.2026.10022.pr5

**Affiliations:** School of Natural Sciences, University of Tasmania, Australia

No accompanying comment.

# Editor report: Recommendation: Explainer: Extaille — R1/PR7

**DOI:** 10.1017/ext.2026.10022.pr7

**Affiliations:** Durrell Institute of Conservation and Ecology, University of Kent, United Kingdom of Great Britain and Northern Ireland

Thanks for taking on board the minor revisions of the reviewers. I’m happy to recommend it be accepted. I do however note a couple of word corrections that could be picked up in the proof. Many thanks for submitting this Explainer, it is much appreciated.

Impact statement - Jargony should be jargon-laden

“These terms (extaille, extation) never established in the academic literature” should be

“These terms (extaille, extation) WERE never established in the academic literature”

Or

“These terms (extaille, extation) never BECAME established in the academic literature”

# Editor report: Decision: Explainer: Extaille — R1/PR8

**DOI:** 10.1017/ext.2026.10022.pr8

**Affiliations:** School of Natural Sciences, University of Tasmania, Australia

No accompanying comment.
